# Ratcheting Behavior of Intervertebral Discs Under Cyclic Compression: Experiment and Prediction

**DOI:** 10.1111/os.12530

**Published:** 2019-10-29

**Authors:** Chun‐qiu Zhang, Tao Zhang, Lilan Gao, Cheng‐fei Du, Qing Liu, Hai‐ying Liu, Xin Wang

**Affiliations:** ^1^ Tianjin Key Laboratory for Advanced Mechatronic System Design and Intelligent Control, School of Mechanical Engineering Tianjin University of Technology Tianjin China; ^2^ National Demonstration Center for Experimental Mechanical and Electrical Engineering Tianjin China

**Keywords:** Constitutive model, Cyclic compression, Intervertebral disc, Ratcheting behavior

## Abstract

**Objective:**

To evaluate the ratcheting behavior of intervertebral discs (IVD) by experiments and theoretical study.

**Method:**

The lumbar spines of sheep were obtained at a local slaughterhouse, and the IVD was processed with upper and lower vertebral bodies (about 5 mm) to ensure the mechanical state of the IVD *in situ*. The ratcheting tests of uniaxial cyclic compression loading for disc samples is carried out using the Electronic Universal Fatigue Testing System at room temperature. The effects of different stress variations, stress rates, as well as different segments on ratcheting behavior of discs were investigated.

**Results:**

The ratcheting strain evolution of lumbar IVD include stages of sharp increase and asymptotic stability. Both the ratcheting strain and ratcheting strain rate increase with an increase of stress variation (*R* = 0.962, *P* = 0.004) but decrease with an increase of the stress rate (*R* = −0.876, *P* = 0.019 ). Compression stiffness increases with an increase of the stress rate (*R* = 0.964, *P* = 0.004 ) or stress variation (*R* = 0.838, *P* = 0.037). Compared with L_5__–__6_, the L_6__–__7_ disc showed less ratcheting strain (*P* = 0.04 ), indicating that the disc at this segment was more resistant to the impact of the ratcheting cycle. In addition, ratcheting strain evolution was predicted using a ratcheting evolution constitutive equation, and the predicted results were in good agreement with experimental data.

**Conclusions:**

The ratcheting behavior occurs in IVD, and this cumulative deformation is consistent with the general ratcheting behavior. The constitutive equation can predict the ratcheting strain evolution of IVD very well. These results are of great significance for the analysis of defects and the development of repair in IVD.

## Introduction

Lumbar degenerative disc disease is one of the most common orthopaedic diseases. It is characterized by loss of water in disc tissues, decrease of internal pressure, rupture of internal structure of nucleus pulposus, and annulus fibrosus. The aggravation of disc degeneration can lead to disc herniation and lumbago, and seriously affect human health. One potential reason is that the spine suffers from long‐term mechanical fatigue. Therefore, it is important to study the mechanical response of discs under cyclic load.

Some research has investigated the mechanical properties of intervertebral discs (IVD). The stress/strain characteristics of different regions of the lumbar IVD under static loading have been tested *in vitro*, and the relationship between the structure of the disc and its mechanical properties have been analyzed. For example, Hollingsworth *et al*.^1^ applied buckling and axial compression to the bovine caudal vertebral motion segment, and tested the biaxial strain state of the fibrous ring, and substituted it into the nonlinear strain energy function to determine the stress state. O'Connell *et al*.^2^ measured the internal deformation of non‐degenerative and degenerative human IVD during flexion and tension under axial compression load, and analyzed the effects of different regions of the disc, loading position, and degeneration on the mechanical properties of lumbar IVD. We used contactless digital image correlation, a contactless full‐field displacement and strain measurement technique, to study the axial strain distribution at different layers of pig lumbar IVD under non‐confining compression and tensile loading conditions. It was found that the strain in the dorsal region is larger than that in the ventral region, and the strain in the lower layer of the ventral region is larger than that in the upper layer, while the strain in the upper layer of the dorsal region is larger^3^, ^4^, ^5^. As for dynamic testing, Wilke *et al*.^6^ measured the stress of the L_4–__5_ disc in adults during their daily activities by inserting a miniature pressure sensor needle from the lateral side of the disc, and found that the stress peak was 0.4–1.3 MPa. Vergroesen *et al*.^7^ loaded the goat lumbar IVD samples under cyclic compression. The effects of long‐term dynamic loading on the intradiscal pressure, the height of the disc, and the compression stiffness were studied, and the relationship between them was investigated. Gooyers *et al*.^8^ studied the peak stress area and the direction in the multilayer fiber ring tissue samples under physiological related loads, and measured the variation characteristics of stress relaxation response at different load axes and regions. The spine is subjected to alternating loads in daily activities such as walking, running, and staircase climbing. The ratcheting behavior of intervertebral discs remains unclear.

Ratcheting behavior is the accumulation of cyclic plastic strain caused by asymmetric cyclic stress. This accumulation leads to material damage and failure, which shows in the daily activities of organisms under cyclic load. Ratcheting behavior of organisms is still a new field in biomechanics research. Gao *et al*.^9^, ^10^ studied, respectively, the ratcheting behavior of cancellous bone and articular cartilage under uniaxial cyclic compression and found that the stress variation and the stress rate were the key factors influencing ratcheting evolution. In terms of a prediction equation, Cai *et al*.^11^, ^12^ proposed a universal ratcheting model (URM) to describe ratcheting strain evolution under arbitrary cyclic stress. It showed that ratcheting deformation is only related to peak stress without considering temperature, creep, and load history.

**Figure 1:**
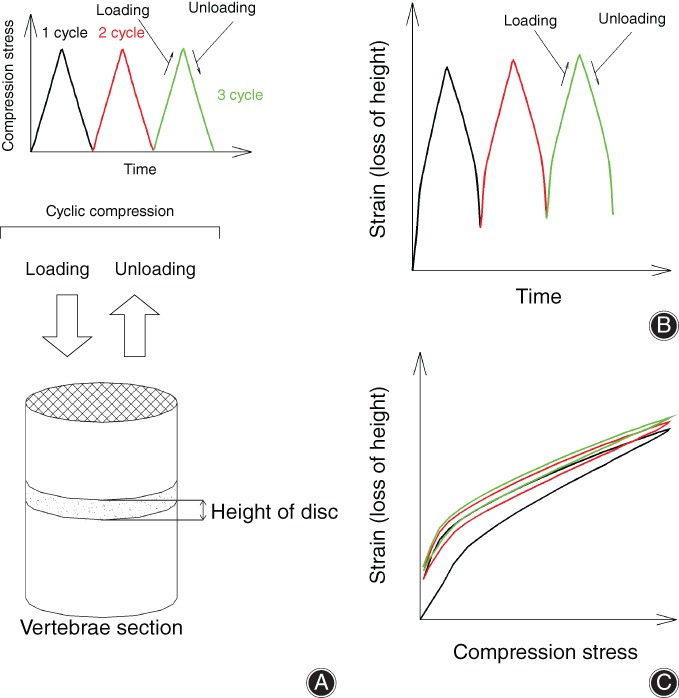
Ratcheting behavior of disc. During the process of loading and unloading, the disc, viscoelastic tissue, produces the accumulation of cyclic strain. The relationship curves between time, compression stress, and strain are given, respectively. (A) Stress control diagram; (B) strain‐time relationship; and (C) strain–stress relationship.

The purpose of the present study is to evaluate the ratcheting behavior of IVD under uniaxial cyclic compression (Fig. 1), the effects of stress variations and stress rates on the ratcheting behavior, and the differences between the ratcheting responses of L_5–6_ and L_6–7_ discs. Furthermore, theoretically, the constitutive equation of the ratcheting evolution is proposed to predict the ratcheting strain evolution of discs.

## Materials and Methods

### *Sample Preparation*

The materials used in this study were taken from the fresh lumbar spine (L_1_–L_7_) of 18‐month‐old sheep at a local slaughterhouse. Each unit of IVD was cut with a bandsaw after the surrounding superfluous muscles and soft tissues were dissected, and facet joints and spinous processes were resected. To ensure the mechanical state of the IVD *in situ*, upper and lower vertebral bodies (approximately 5 mm) were retained for each disc to be fixed by clamps in the experiment. The elliptic area of the IVD was obtained by measuring the length and width of the disc and multiplied by the coefficient *k* = 1.01 (the value of *k* was estimated from the pre‐experiment), from which the disc area *S* = 394 ± 13 mm^2^ was obtained, and the height *h* = 3.98 ± 0.12 mm^13^.

### *Ratcheting Testing*

**Figure 2:**
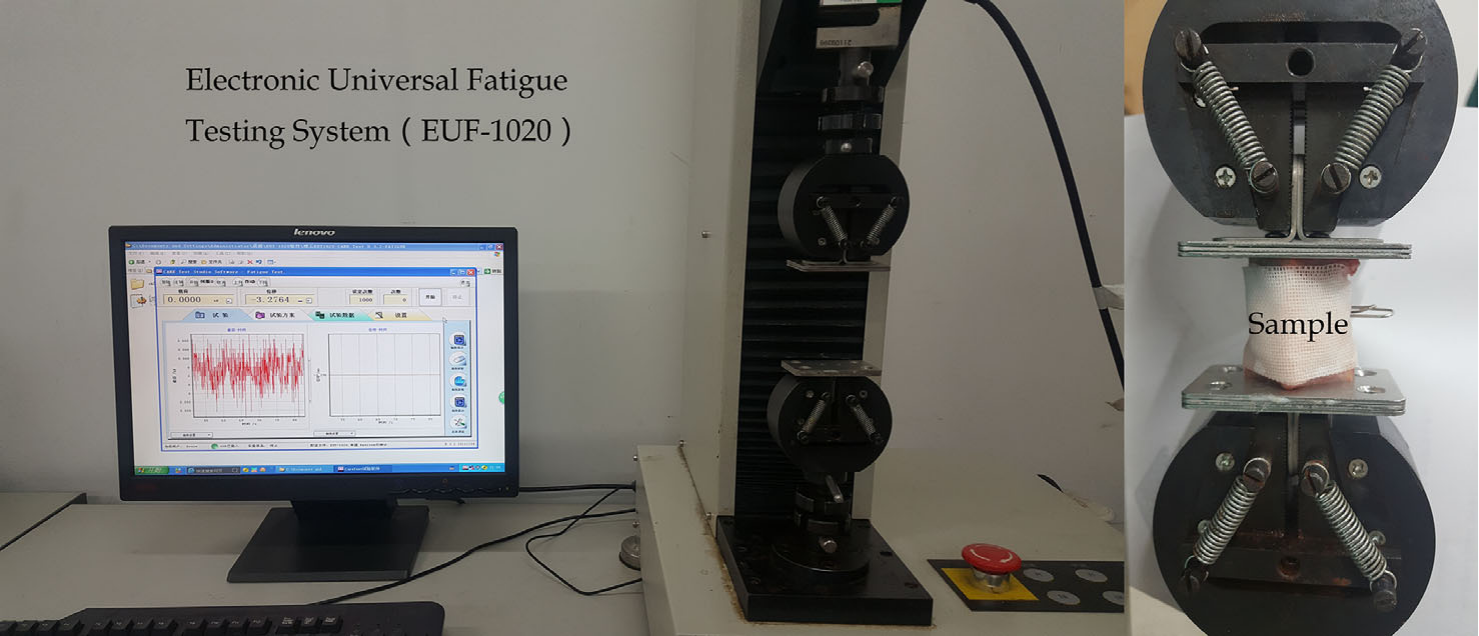
Testing setup and sample. Each disc sample is fixed by clamps to ensure the mechanical state of the intervertebral discs *in situ*. During the test, the saline is replenished to the sample at intervals to prevent moisture from evaporating in the air.

The experiment was carried out at room temperature (26 °C). To avoid the evaporation of water in the air during loading, the gauze, containing sufficient saline, was wrapped around the disc samples, as shown in Fig. 2, and the saline was continuously replenished during the experiment. Each condition was repeated at least three times to ensure the reproducibility. The ratcheting tests of uniaxial cyclic compression loading for disc samples was carried out using the Electronic Universal Fatigue Testing System (EUF‐1020). This system can automatically provide displacement and pressure data, through which the engineering stress and strain can be calculated. The detailed loading condition is shown in Table 1.

**Table 1:** Loading condition

Specimen type	Stress variation (Δσ, MPa)	Stress rate (MPa/s)
L_1_–L_4_	0.59	0.13
L_1_–L_4_	0.59	0.4
L_1_–L_4_	0.59	1.18
L_4_–L_7_	0.59	0.59
L_4_–L_7_	0.88	0.59
L_4_–L_7_	1.18	0.59
L_4_–L_7_	1.76	0.59
L_1_–L_4_	1.76	0.4
L_1_–L_4_	1.76	0.59
L_1_–L_4_	1.76	1.18

The stress variations of 0.59–1.18 MPa are derived in the daily physiological loads on human L_4–__5_ discs^6^, ^14^. In this study, because the stress valley is always 0 MPa, the stress variation is consistent with the peak stress. The stress rate is determined by the frequency of human daily activities.

### *Parameters for Measurement*

**Figure 3:**
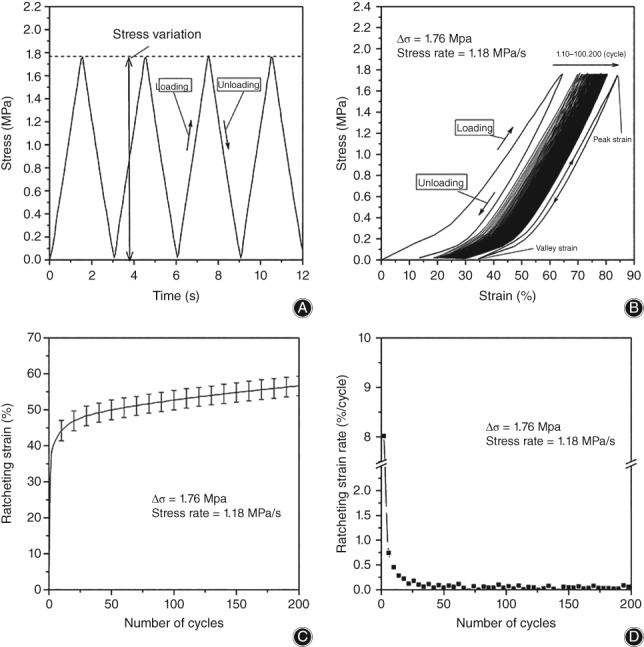
Uniaxial cyclic compression ratcheting tests of intervertebral disc. With the increase of number of cycles, the stress–strain hysteresis loop tends to be dense. The ratcheting strain increases. In contrast, the ratcheting strain rate decreases: (A) stress control diagram; (B) stress–strain relationship, 200 loading cycles; (C) ratcheting strain evolution; and (D) ratcheting strain rate evolution.

To reflect the accumulative deformation of ratcheting behavior, ratcheting strain and ratcheting strain rate are defined, and are derived from the following formulas:

(1) $$\varepsilon_{r}=\frac{\varepsilon_{\min}+\varepsilon_{\max}}{2}$$

(2) $${\dot{\varepsilon }}_{r}=\frac{d\varepsilon_{r}}{\mathrm{dN}},$$

where *ε*_*r*_ is ratcheting strain; *ε*_*min*_ and *ε*_*max*_ are the valley and peak values of strain, respectively (as show in Fig. 3B, the minimum and maximum of axial strain in a cycle, respectively); ${\dot{\varepsilon }}_{r}$ is the ratcheting strain rate, the increment of ratcheting strain in unit number of cycles; and *N* is the number of cycles.

#### *Ratcheting Strain (ε_r_)*

Ratcheting strain is the average of the minimum strain and the maximum strain in each circle, which is used to describe the deformation of materials under cyclic loading. Ratcheting behavior is the accumulation of cyclic plastic strain caused by asymmetric cyclic stress. This accumulation leads to material damage and failure, which shows in the daily activities of organisms under cyclic load.

#### *Ratcheting Strain Rate (* ${\dot{\varepsilon }}_{r}$ *)*

The ratcheting strain rate is the increment of ratcheting strain per unit cycle. The clinical significance of the ratcheting strain and the ratcheting strain rate can describe the deformation law based on time of IVD under different activities.

### *Statistical Analysis*

The ratcheting behavior and stress rate, stress variation, as well as different segments among L_5_–L_7_ were analyzed, respectively, by ANOVA analysis. The correlation between the ratcheting behavior and the stress rate, and stress variation, is tested by Pearson relevant analysis. The prediction and experimental data are assessed by coefficient of determination.

## Result

### *Ratcheting Behavior of Intervertebral Discs Under Uniaxial Cyclic Load*

The ratcheting test is performed with cyclic compression for the disc. The typical triangular wave load condition is applied as shown in Fig. 3A. Figure 3B shows the stress–strain curve of the ratcheting test for a disc with stress variation of 1.76 MPa and a stress rate of 1.18 MPa/s, including the process of loading compression and unloading recovery. It shows that the stress–strain hysteretic loop is looser in the initial cyclic load. With the increase in the number of cycles, the hysteretic curves tend to be dense, and the hysteretic loop is not closed at the 200th cycle.

The relationship between the ratcheting strain and the number of cycles is shown in Fig. 3C. It reveals that the ratcheting strain increases sharply at the beginning of cyclic loading. Especially in the first five cycles, ratcheting strain increases by 40%. When the number of cycles reaches 30, ratcheting strain gradually increases at a relatively stable and slow speed, and the duration of this stage is longer. Figure 3D shows the relationship between the ratcheting strain rate and the number of cycles. The ratcheting strain rate decreases sharply in the first few cycles. With the increase of the number of cycles, the ratcheting strain rate decreases slowly and steadily, but it is always greater than 0.

### *Effect of Stress Variation on Ratcheting Behavior of Intervertebral Discs*

**Figure 4:**
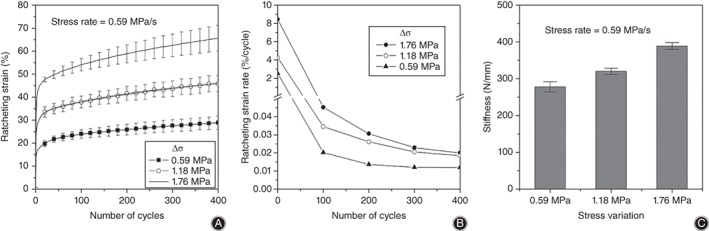
Ratcheting behavior with different stress variations. Ratcheting strain, ratcheting strain rate, and compression stiffness increase with the increase of stress variation: (A) Ratcheting strain evolution; (B) ratcheting strain rate evolution; and (C) compression stiffness at the 400th cycle.

Stress variation has a great influence on the ratcheting behavior of discs (Fig. 4), where the stress variation is 0.59, 1.18, and 1.76 MPa, respectively, and the stress rate is 0.59 MPa/s. As shown in Fig. 4A, the ratcheting strain increases with the increase of stress variation because the stress rate is constant (*R* = 0.962, *P* = 0.004, ratcheting strain is selected at 400th cycle). Figure 4B shows that the ratcheting strain rate increases with the increase of stress variation.

Figure 4C shows the compression stiffness of different stress variations at the 400th cycle. It is found that the larger the stress variation, the greater the compression stiffness (*R* = 0.964, *P* = 0.004).

### *Effect of Stress Rate on the Ratcheting Behavior of Intervertebral Discs*

**Figure 5:**
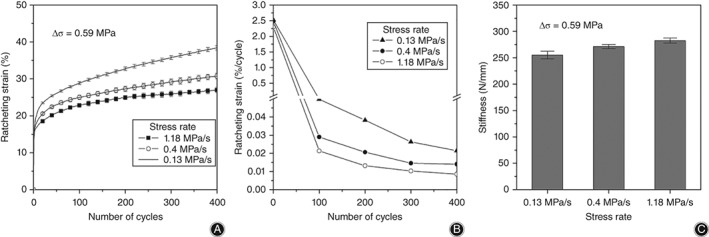
Ratcheting behavior with different stress rates. Ratcheting strain and ratcheting strain rate decrease with the increase of stress rate. In contrast, the compression stiffness increases: (A) Ratcheting strain evolution; (B) ratcheting strain rate evolution; and (C) compression stiffness at the 400th cycle.

The ratcheting behavior corresponding to different stress rates is not consistent in Fig. 5. Here, the stress rate is 0.13, 0.4, and 1.18 MPa/s, respectively, and the stress variation is 0.59 MPa. As shown in Fig. 5A, when the stress variation is constant, the ratcheting strain decreases with an increase of the stress rate (*R* = −0.876, *P* = 0.019 < 0.05, ratcheting strain is selected at the 400th cycle), which indicates that ratcheting behavior of discs is rate‐dependent. In addition, the ratcheting strain difference between stress rates of 0.13 and 0.4 MPa/s is much larger than that between 0.4 and 1.18 MPa/s. Figure 5B shows the effects of the stress rate on the ratcheting strain rate, and the ratcheting strain rate decreases with an increase of the stress rate.

Figure 5C shows the compression stiffness of different stress variations at the 400th cycle. It can be found that the compression stiffness increases with an increase of the stress rate (*R* = 0.838, *P* = 0.037 ).

### *Ratcheting Behavior of L_5–__6_* *and L_6–__7_* *Discs at Different Stress Variations*

**Figure 6:**
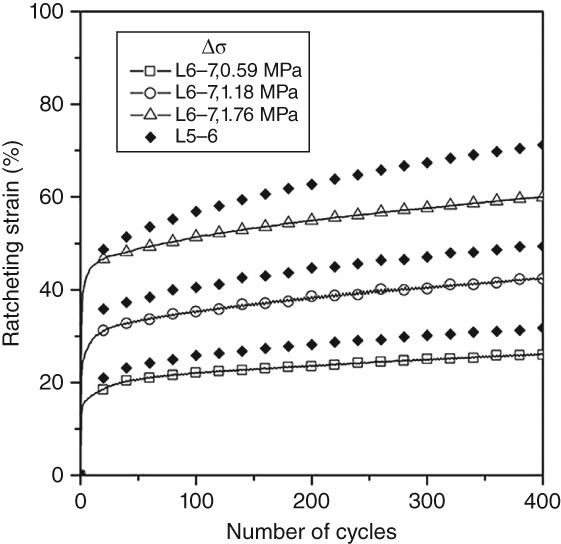
Ratcheting strain evolution. Ratcheting strain of the L6–L7 disc is always less than the L5–L6 disc under different loading conditions.

Figure 6 shows the ratcheting strain evolution of L_5–__6_ and L_6–__7_ discs, where the stress variation is 0.59, 1.18, and 1.76 MPa, respectively. It should be noted that unlike the human body, the lumbar spine of the selected sheep has seven segments, so L_6–__7_ is located at the end. It is found that the ratcheting strain of L_6–__7_ is always smaller than that of L_5–__6_, regardless of the size of the stress variation (*P* = 0.04, ratcheting strain is selected at the 400th cycle), which indicates that this segment is more resistant to cyclic loading.

### *Ratcheting Evolution Constitutive Model of Intervertebral Discs*

To study the ratcheting strain evolution of discs, a one‐dimensional stress parameter constitutive equation for predicting the ratcheting strain evolution of IVD has been developed based on the URM. It has been established to understand the ratcheting strain evolution of metallic materials, and is used to describe the cumulative effect of ratcheting deformation of discs under uniaxial cyclic compression. The improved model is as follows:

(3) $$X_{r}=K\cdot X_{\mathrm{SR}}\cdot \left(1-\alpha N^{\beta }\right)$$

(4) $$X_{\mathrm{SR}}=k_{r}\cdot \left(e_{r}-e_{\mathrm{rth}}\right),$$

where *X*_*r*_ (*ε*_*r*_) is ratcheting strain; *e*_*r*_ is ratcheting stress (peak stress, one‐dimensional control parameter); *X*_*SR*_ is saturated ratcheting strain (ratcheting strain reaches or approaches a saturated state); *α* and *β* are ratcheting evolution parameters, which are generally monotonously dependent on ratcheting stress, respectively; *e*_*rth*_ is the ratcheting threshold; *k*_*r*_ is the ratcheting coefficient; *N* is number of cycles; and *K* is a material coefficient (*k*_*r*_, *e*_*rth*_, and *K* are deduced from experimental data).

The experimental data is regressed to obtain the corresponding *K* value, as shown in Table 2.

**Table 2:** Material coefficient *K* values corresponding to different stress variations and rates

Stress rate (MPa/s)	Stress variation (Δσ, MPa)								
	0.59			1.18			1.76		
0.13	0.0107	0.0108	0.0107						
0.4	0.0092	0.009	0.0092				0.009	0.0091	0.0091
0.59	0.0093	0.0102	0.0093	0.0091	0.0104	0.0091	0.0092	0.0104	0.0091
							0.009	0.0092	0.009
1.18	0.0089	0.0091	0.0088				0.0093	0.0093	0.0093

**Figure 7:**
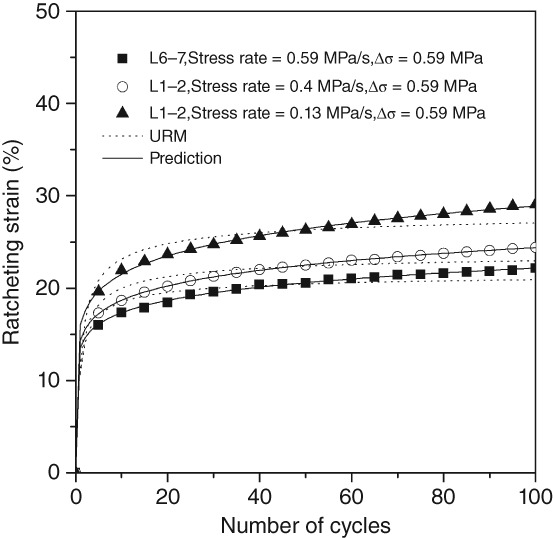
Simulating for experimental data of ratcheting strain using the universal ratcheting model and the modified model (*K* = 0.01). Visually. the modified model fits the experimental data well.

It is found that the *K* value is close to 0.01, whether the stress variation or the stress rate is large or small, and the correlation coefficient between the new model and the experimental data is as high as 0.99 by introducing *K* = 0.01 into Equation (3). Figure 7 shows the simulation of experiments under different conditions by URM and this model, respectively. It is obvious that this model can well describe the ratcheting strain evolution of discs.

**Figure 8:**
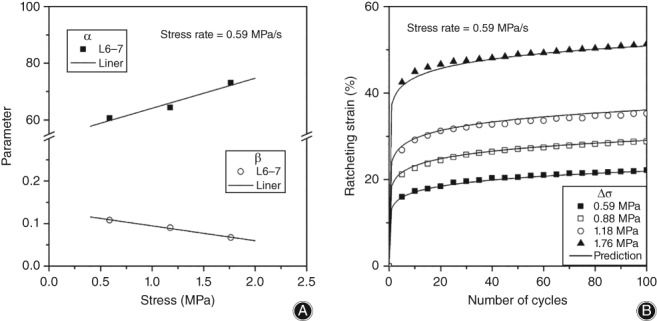
Prediction results for ratcheting evolution. The experimental data shows that the evolution parameters *α* and *β* depend monotonously on ratcheting stress. Equations 5, 6 are obtained by determining the linear relationship between evolution parameters and ratcheting stress. After determining the ratcheting parameters, the ratcheting strain evolution of the disc is predicted, and the predicted results are well matched with the experimental data: (A) relationship between evolution parameters and ratcheting stress, respectively and (B) prediction of ratcheting strain evolution.

The prediction result of ratcheting evolution of L_6–__7_ using this model (*K* = 0.01) is shown in Fig. 8. As shown in Fig. 8A, evolution parameters are monotonically dependent on ratcheting stress by regression analysis, and are expressed as follows:

(5) $$\alpha =53.565+10.571\cdot e_{r}$$

(6) $$\beta =0.13-0.035\cdot e_{r}$$

Figure 8B shows that the predicted results of ratcheting strain evolution using this model are in good agreement with the experimental data.

## Discussion

The ratcheting behavior of IVD need to be studied because the human spine suffers from long‐term mechanical fatigue. In daily activities, the discs in humans are subjected to dynamic loads, which can result in a continuous accumulation of deformation due to the alternation of inhomogeneous fluid exudation and imbibition in the matrix^7^, ^15^. The accumulation of deformation can explain why the height of the human body is shorter at night than in the morning due to the ratcheting effect of the intervertebral disc. As the load progresses, the alternating deformation in the disc gradually overlaps with the damage strain range, resulting in injury of the vertebral column. For example, for soldiers or athletes, prolonged excessive spinal deformation can lead to premature degeneration of their discs.

The ratcheting behavior of IVD under uniaxial cyclic compression is studied by experiment and prediction. As shown in Fig. 3B, strain (peak and valley strain) increases with the increase of cycles, but strain range (absolute difference of peak and valley strain) decreases with the increase of cycles, and hysteretic loops change from sparse to dense, which is closely related to the fluid flow in the porous structure of disc and soft tissue deformation^16^, ^17^. On the one hand, insufficient hydration during cyclic load reduces fluid in the disc and the disc cannot return to its original height when unloaded, which leads to the continuous decrease of disc height during loading. On the other hand, due to the viscoelastic mechanical properties of the soft tissues in the disc, residual stress is produced by the continuous hardening of the soft tissues under stress, so the soft tissue, IVD, cannot be restored to its original status in a short period of time. Typical ratcheting strain evolution includes three stages: transient, quasi‐steady, and tertiary^18^, ^19^. During the third stage, the ratcheting plastic accumulation may eventually lead to the failure of the materials. As shown in Fig. 3C. The ratcheting strain of the disc increases sharply in the first few cycles of cyclic load, then maintains a steady and slow growth rate. In this test, the final(tertiary) stage was invisible because only 200 cycles were performed under cyclic load.

Stress variation and the stress rate (frequency) are the representative factors for discs in load‐bearing, walking, and running. It is believed that the stress rate and stress variation can affect the ratcheting behavior of materials. Figures 4 and 5 show that both the ratcheting strain and the ratcheting strain rate increase with an increase of stress variation but decrease with an increase of the stress rate, which concurs with the results of Gao *et al*.^9^, ^10^. Compression stiffness increases with an increase of stress variation or the stress rate. In general, with an increase of stress variation, the fluid in the nucleus pulpous of discs is gradually squeezed into the annulus fibrosus, which increases the weight‐bearing area and requires a greater load in compression, thus stiffness. When the stress rate increases, because of the porous structure (fluid flow) in the disc, excessive load speed causes some fluids to be trapped in the soft tissue and unable to seep out in a short period of time. Fluids play a major role in resisting load, resulting in smaller ratcheting strain and larger compression stiffness.

A lot of work has been undertaken to investigate the ratcheting evolution constitutive model, but most of the theoretical equations are very complex, and the parameters of which require high debugging skills to be solved iteratively^20^, ^21^. Cai *et al*.^11^ found that the monotonic ratcheting strain evolution law of materials conformed to the power function if the effects of temperature and load history were neglected, from which the ratcheting evolution model URM was controlled by one‐dimensional parameter. Based on URM, we add the material coefficient *K* to construct a one‐dimensional parameter‐controlled ratcheting evolution model of IVD. A large number of experimental data fit to determine *K* = 0.01, and the correlation coefficient between this model and experimental data is 0.99. The experimental results show that there is a good monotonic dependence between stress variation and saturated ratcheting strain in this test. Therefore, we simplify the relationship between ratcheting stress and saturated ratcheting strain into Equation (4), which is consistent with the results of Cai *et al*. There is, indeed, an interval in which the ratcheting stress has a linear relationship with the saturated ratcheting strain. To validate the model, ratcheting experiments with stress variations of 0.59, 1.18, and 1.76 MPa were used, and the ratcheting parameters were determined as shown in Fig. 8. Ratcheting experiments with stress variation of 0.88 MPa compared with the predicted result, and there is good agreement between the predicted results and the experimental data.

In this paper, the model can predict the transient and quasi‐steady state of the ratcheting strain evolution of IVD very well. Similar to URM, it also needs only 3–4 ratcheting experimental data to establish. However, the shortcoming is that because the stress variations selected in this test are too large, ratcheting stress and saturated ratcheting strain only show a linear relationship^22^.

### *Conclusion*

The ratcheting behavior of IVD is studied experimentally and predicted theoretically. It is found that ratcheting strain increases sharply under cyclic compression at the beginning, and then with the increase in the number of cycles, ratcheting strain evolves into a stable state, and this upward trend becomes slow and steady, which conforms to the general ratcheting strain evolution. The ratcheting strain and ratcheting strain rate increase with an increase of stress variation but decrease with an increase of the stress rate. The ratcheting strain of the L_6–__7_ disc was smaller than that of L_5–__6_ with an increase of number of cycles. The compression stiffness of discs increases with an increase of stress variation or the stress rate. In addition, by introducing the URM model and adding the material coefficient *K* = 0.01, the predictive constitutive equation for the ratcheting strain evolution of the disc is obtained. These results are of great significance for the analysis of defects and development of repair of IVD.
